# Research on End-Effector Position Error Compensation of Industrial Robotic Arm Based on ECOA-BP

**DOI:** 10.3390/s25020378

**Published:** 2025-01-10

**Authors:** Wenping Xiang, Junhua Chen, Hao Li, Zhiyuan Chai, Yinghou Lou

**Affiliations:** 1 College of Science and Technology, Ningbo University, Ningbo 315300, China; 2211170028@nbu.edu.cn (W.X.); chenjunhua@nbu.edu.cn (J.C.); lihao1@nbu.edu.cn (H.L.); 2211170016@nbu.edu.cn (Z.C.); 2 Faculty of Mechanical Engineering and Mechanics, Ningbo University, Ningbo 315211, China

**Keywords:** industrial robotic arm, rigid–flexible coupling, error compensation, ECOA-BP neural network

## Abstract

Industrial robotic arms are often subject to significant end-effector pose deviations from the target position due to the combined effects of nonlinear deformations such as link flexibility, joint compliance, and end-effector load. To address this issue, a study was conducted on the analysis and compensation of end-position errors in a six-degree-of-freedom robotic arm. The kinematic model of the robotic arm was established using the Denavit–Hartenberg (DH) parameter method, and a rigid–flexible coupled virtual prototype model was developed using ANSYS and ADAMS. Kinematic simulations were performed on the virtual prototype to analyze the variation in end-effector position errors under rigid–flexible coupling conditions. To achieve error compensation, an approach based on an Enhanced Crayfish Optimization Algorithm (ECOA) optimizing a BP neural network was proposed to compensate for position errors. An experimental platform was constructed for error measurement and validation. The experimental results demonstrated that the positioning accuracy after compensation improves by 75.77%, fully validating the effectiveness and reliability of the proposed method for compensating flexible errors.

## 1. Introduction

As a highly flexible and precise automated device, robotic arms have demonstrated broad application potential across multiple fields. With the continuous advancement of artificial intelligence, machine vision, and sensor technologies, the application range of robotic arms has expanded from traditional industrial automation to industries such as healthcare, services, logistics, and agriculture, gradually transforming production modes and workflows across various sectors. In the field of industrial manufacturing, robotic arms have become a core component of automated production lines, widely applied in automotive manufacturing, electronic assembly, welding, and painting processes. Through high-precision and high-efficiency operations, robotic arms efficiently complete repetitive and high-intensity tasks, significantly improving productivity and ensuring consistency in product quality [1,2]. In the medical field, the application of robotic arms has shown significant innovation potential, especially in surgical procedures. By providing higher operational precision and flexibility, robotic arms have advanced minimally invasive surgeries, significantly reducing patient trauma and accelerating recovery [3,4]. Traditional crack detection methods typically rely on manual inspections or fixed detection equipment, which suffer from low efficiency, poor accuracy, and safety risks. With the development of robotic arm technology, robotic arms have gradually become an effective alternative for crack detection [5].

The application of robotic arms in various fields not only improves production efficiency and accuracy and reduces labor costs but also drives technological innovation and industry development. As artificial intelligence, sensor technology, and robotic arm control systems continue to advance, the applications of robotic arms will further expand, having a profound impact on more industries and continuing to transform traditional production methods and workflows.

The “Industry 4.0” initiative and China’s “Made in China 2025” strategy both emphasize the importance of intelligent manufacturing and automation. As key technologies for achieving these objectives, serial robotic arms play a crucial role in driving the transformation and upgrading of the manufacturing industry. While serial robotic arms offer high repeatability in positioning accuracy (up to 0.02 mm), their absolute positioning accuracy typically ranges from 1 mm to 3 mm, which falls short of the requirements for high-end manufacturing applications [6]. Therefore, improving absolute positioning accuracy is of paramount importance for both the manufacturing industry and automation systems. Enhancing accuracy not only improves product quality, production efficiency, and system stability, but also reduces costs, enhances safety, and fosters technological innovation and intelligent development.

Extensive research has been conducted worldwide to improve the precision of serial robotic arms. For example, Li et al. [7] proposed a real-time trajectory error compensation method using laser trackers to measure the robot’s terminal position in real time, achieving absolute positioning errors of less than 0.06 mm and trajectory position errors of less than 0.15 mm. Zou et al. [8] introduced a hierarchical calibration method that combines kinematic calibration and spatial interpolation to effectively reduce absolute positioning errors. Ren et al. [9] investigated gravity error identification and compensation methods for a self-developed six-degree-of-freedom joint robot, significantly improving the positioning accuracy of the end-effector. Lu et al. [10] proposed an Incremental Extreme Learning Machine (IELM) model to improve the positioning accuracy of robots, which was validated on the KUKA KR160 industrial robot. The experimental results showed an 86% improvement in milling robot accuracy after compensation. Chen et al. [11] developed a spatial IDSW interpolation algorithm for pose error compensation and verified its effectiveness through comparative simulations. Gao et al. [12] introduced a spatial error similarity-based method incorporating distance and direction for positioning error prediction and compensation, demonstrating substantial error reductions across all axes compared to traditional inverse distance weighting methods. Jiao et al. [13] proposed a graded compensation strategy that couples kinematic and load flexibility errors for precise robotic control in machining tasks. He et al. [14] developed a residual compensation method based on error similarity to enhance end-effector positioning accuracy. Gao et al. [15] employed a BP neural network-based modeling and error compensation method for AACMMs, achieving a 97% error reduction. Yin et al. [16] proposed an iterative error compensation technique for stone carving robotic manipulators (SCRMs), accounting for the coupling between compensation and deformation. This method was validated on the KUKA-240-2900 SCRM system, demonstrating its feasibility and effectiveness. However, most of the above studies focus on addressing a single type of error or rely on precise mathematical models for compensation. These approaches often face certain limitations when dealing with nonlinear errors in robotic systems.

To address these challenges, this paper proposed an innovative integrated error compensation method for the end-effector of a serial manipulator based on the ECOA-BP neural network. Unlike traditional error compensation methods, this approach constructed the kinematic model of the serial manipulator and used ADAMS 2020 software for rigid–flexible coupling simulations to obtain more realistic error data. An Enhanced Crayfish Optimization Algorithm (ECOA) is then used to optimize the BP neural network for error model training, achieving offline error compensation based on data. This method not only addresses complex nonlinear errors caused by link and joint flexibility deformation and load-induced errors but also continuously optimizes the neural network’s learning process, enabling the compensation system to effectively correct various types of errors, even without an accurate mathematical model. Compared to existing single-error compensation methods, the proposed method demonstrated significant advantages in improving end-effector positioning accuracy and enhancing compensation adaptability, with improved flexibility and stability.

The proposed end-effector error compensation method for the serial manipulator, based on the ECOA-BP neural network, not only integrates the ECOA-optimized BP neural network for comprehensive error compensation but also adapts to the multi-source errors and nonlinear characteristics of complex systems, overcoming the limitations of traditional methods under high-precision requirements. This method provides a novel approach and technical pathway for improving the positioning accuracy of serial manipulators and holds promising application prospects.

## 2. Kinematic Model of the Robotic Arm

### 2.1. Establishment of the DH Coordinate System

**Figure 1:**
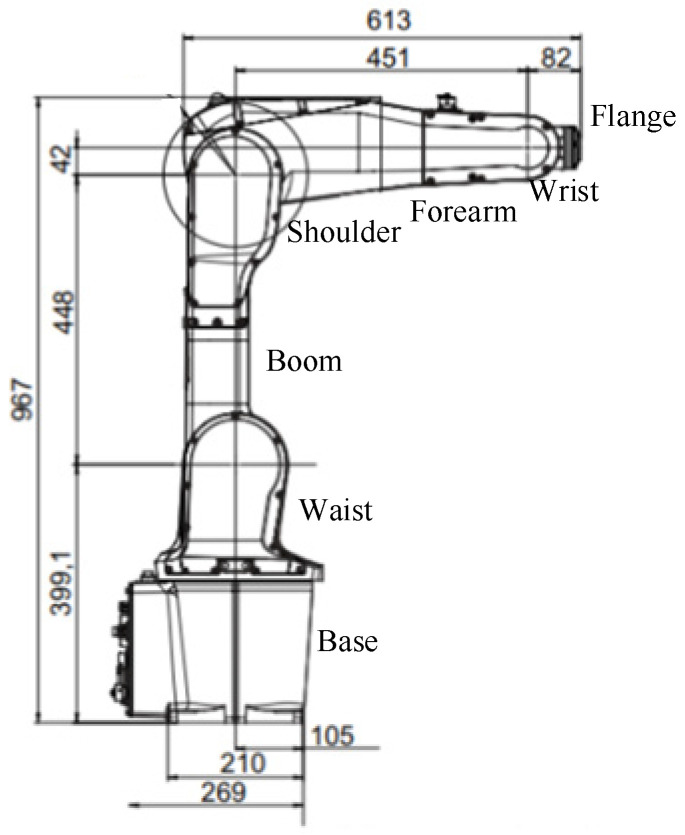
The IRB1200 robotic arm structure.

**Figure 2:**
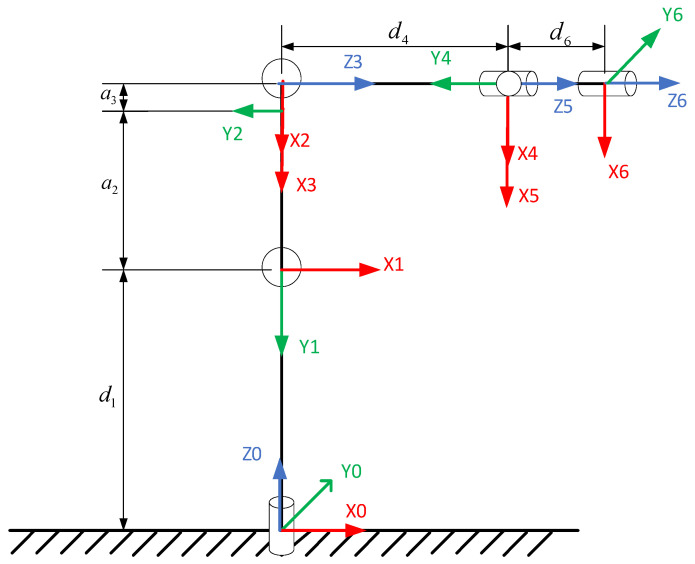
Link coordinate system of the IRB1200 robotic arm.

The IRB1200 robotic arm is a six-degree-of-freedom serial robotic arm equipped with six rotary joints. Its structure consists of a base, waist, upper arm, shoulder, forearm, wrist, and flange, as shown in Figure 1. The robotic arm has a maximum payload capacity of 5 kg, repeatability of 0.025 mm, and an overall weight of 54 kg. The Denavit–Hartenberg (DH) convention is applied to establish the link coordinate system of the IRB1200 robotic arm [17,18,19], as illustrated in Figure 2. The corresponding DH parameters are summarized in Table 1.

The DH parameters provide a concise representation of the robotic arm’s structure and pose. When analyzing factors affecting the end-effector’s pose accuracy, these factors can be translated into structural influences, represented by deviations in the DH parameters. By utilizing an error model based on these DH parameter deviations, the pose error at the end-effector can be calculated.

### 2.2. Forward Kinematic Modeling of the Robotic Arm

Based on the link coordinate system {i}, the relative position of the link coordinate system {i−1} can be described using four homogeneous transformations. Utilizing the chain rule for coordinate system transformations, the transformation matrix from coordinate system {i−1} to coordinate system {i} can be expressed as follows [20,21]:

(1) Tii−1=Rot(Z,θi)Trans(0,0,di)Trans(ai,0,0)Rot(X,αi)=cθisθi00sθicθi000010000110000100001di0001100ai01000010000110000cαi−sαi00sαicαi00001

(2) Tii−1=cθi−sθicαisθisαiaicθisθicθicαi−cθisαiaisθi0sαicαidi0001

where c stands for cos and s stands for sin.

The DH parameters presented in Table 1 can be combined with the transformation matrix described above to obtain the relationship between each link:

T10=cθ10sθ10sθ10−cθ10010d10001

T21=cθ2−sθ20a2cθ2sθ2cθ20a2sθ200100001

(3) T32=cθ30sθ3a3cθ3sθ30−cθ3a3sθ301000001  T43=cθ40−sθ40sθ40cθ400−10d40001

T54=cθ50sθ50sθ50−cθ5001000001  T65=cθ6−sθ600sθ6cθ600001d60001

By multiplying the transformation matrices between adjacent joints, the overall transformation matrix of the robotic arm can be derived. The equation is given as follows:

(4) T60=T10T21T32T43T54T65=nxoxaxpxnyoyaypynzozazpz0001

In the above formula, n represents the direction vector of the robot’s end-effector along the *X*-axis in the base coordinate system; o represents the direction vector along the *Y*-axis; a represents the direction vector along the *Z*-axis; and p represents the coordinates of the robot’s end-effector in the base coordinate system.

#### Robot Arm Model Validation

**Figure 3:**
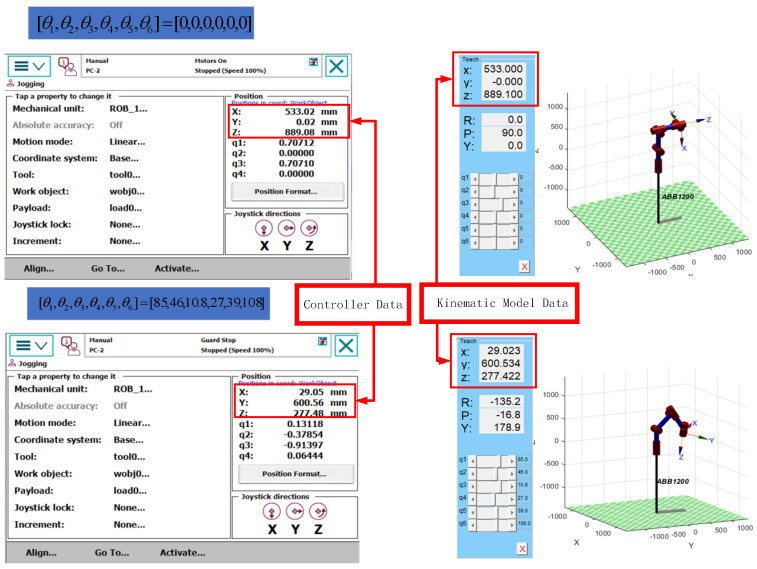
Validation of the IRB1200 robotic arm model.

To verify the accuracy of the established robotic arm model, the IRB1200 robotic arm was controlled via the teach pendant to reach a predetermined target position. The corresponding joint angles for this target position were recorded and then input into the theoretical model of the robotic arm. Subsequently, the end-effector position coordinates for both sets of joint angles were compared: one set corresponding to the initial state [0, 0, 0, 0, 0, 0] and the other set corresponding to the target state [85, 46, 10.8, 27, 39, 108]. As shown in Figure 3, by comparing the end-effector position coordinates corresponding to these two sets of joint angles, it was confirmed that the end-effector positions matched, thereby validating the correctness of the robotic arm model.

### 2.3. Solving the Robot’s Workspace

**Figure 4:**
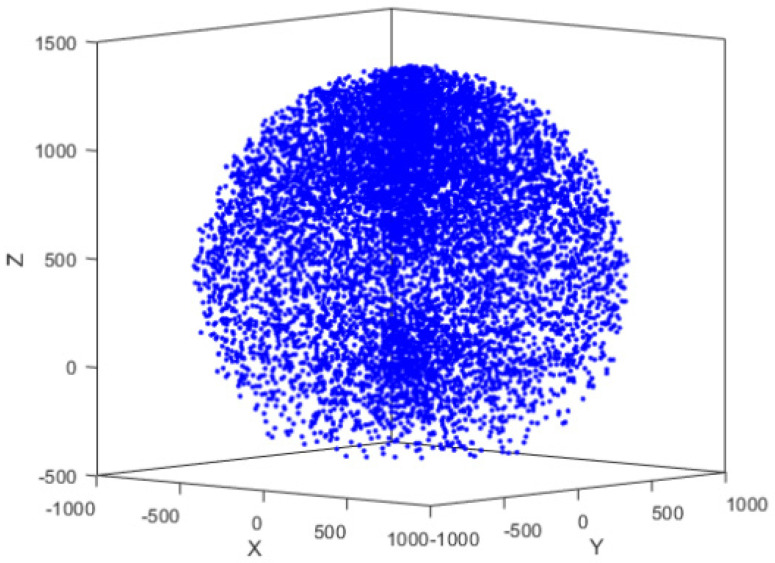
Workspace of the robotic arm.

When performing error compensation for a robotic arm, data collection must effectively cover the entire workspace of the arm. Therefore, it is essential to determine its workspace. The Monte Carlo method [22,23] is a technique that approximates solutions to complex problems through random sampling. To determine the workspace of a 6-degree-of-freedom robot, the Monte Carlo method generates a large number of random joint angle combinations. Using forward kinematics calculations, the reachable positions of the robot’s end-effector are obtained, thereby defining the workspace. By conducting simulations in MATLAB, the workspace of the IRB1200 industrial robotic arm can be determined. The workspace of the robotic arm is shown in Figure 4.

## 3. Establishing the Virtual Prototype Model

To ensure that the samples accurately reflect the original state of the robotic arm’s positioning error, it is essential to effectively cover the entire workspace of the robotic arm during data collection. However, existing measurement methods, such as laser trackers and coordinate measuring machines, encounter difficulties in fully covering all working regions of the robotic arm. To address this issue, this paper proposed a method for collecting error data using a virtual prototype, which enables data collection across the full workspace of the robotic arm. This approach mapped the robotic arm’s actual motion states onto a virtual prototype model, allowing direct sampling within the virtual environment.

**Figure 5:**
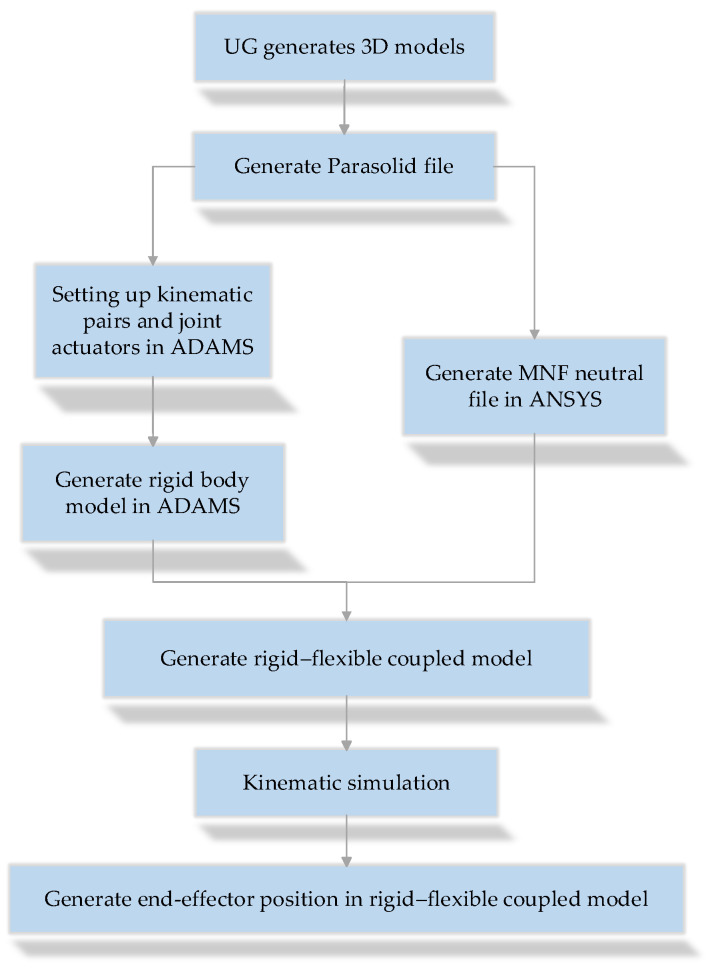
Process of generating the rigid–flexible coupled prototype.

The construction of the virtual prototype began with creating a 3D geometric model of the robotic arm using UG 12.0 software and then exporting the model as a Parasolid format file. Subsequently, the Parasolid file was imported into the ADAMS platform, where motion pairs were defined, and joint drive modes were configured. Using ANSYS 2020 R2 software, a Modal Neutral File (MNF) was generated, which enables coupling between the rigid body model and flexible components, forming a rigid–flexible coupled prototype model. Based on this model, simulation analysis was conducted, ultimately obtaining the coordinates of the robotic arm’s end-effector. The process of generating the prototype for rigid–flexible coupling is shown in Figure 5.

### 3.1. Creation of Flexible Linkage and Flexible Joints

**Figure 6:**
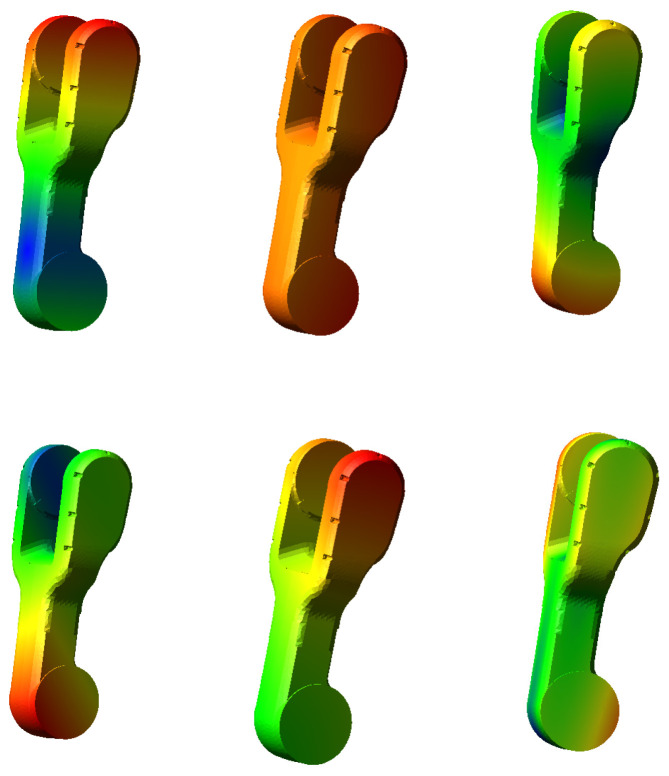
Mode shape cloud diagrams of the flexible upper arm from the 7th to 12th order.

Under the influence of the self-weight of the links and external loads, the links and joints of a serial robotic arm undergo deformation. Therefore, when analyzing end-effector positioning errors, it is essential to account for the flexibility of the links and joints, allowing them to exhibit elastic deformation under force. This approach more accurately reflects the actual behavior of the robotic arm. In a serial robotic arm, longer links experience more pronounced deformation under force, while shorter links are less affected. As a result, only the upper arm and shoulder were treated with flexibility adjustments. Figure 6 presents the 7th to 12th mode shape cloud diagrams of the flexible upper arm.

**Figure 7:**
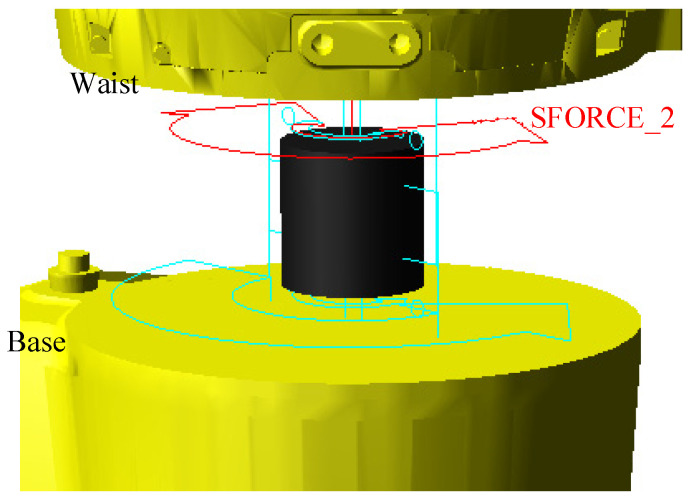
Schematic diagram of the flexible joint 1 model.

In a real serial robotic arm, due to the insufficient rigidity of the actuators and transmission mechanisms, joint flexibility leads to additional angular increments. These angular increments are transmitted through the links of the robotic arm to the end-effector, resulting in significant positional deviations. To improve simulation accuracy and ensure the model aligns with real-world conditions, it is necessary to account for the flexibility of the joints in the serial robotic arm. In this paper, the joints were represented as torsional springs. In ADAMS, a torque function was introduced at the joints to model this flexibility. Taking the joint between the base and waist as an example, a rotational pair and driving function were added at the input end of the base’s rotating axis, while a rotational pair and joint torque were introduced at the output end of the waist to drive its rotation. The flexible joint 1 model is shown in Figure 7.

### 3.2. Rigid–Flexible Coupled Virtual Prototype Model Motion Simulation

**Figure 8:**
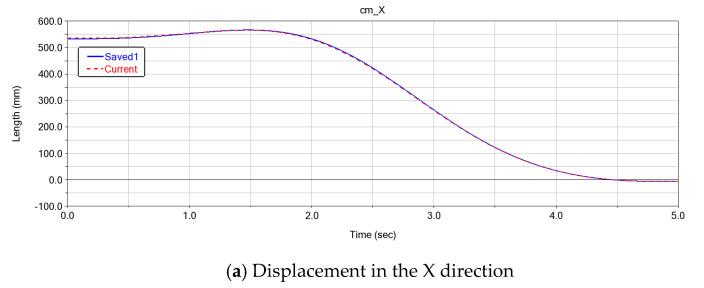

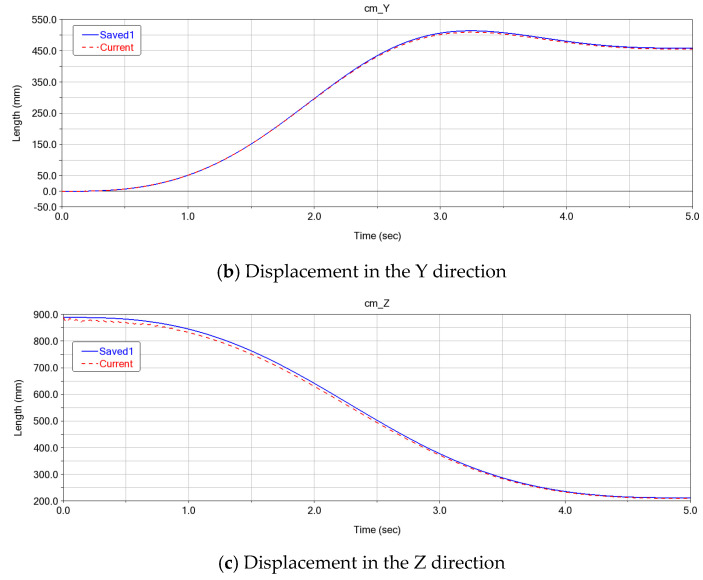
Displacement curves in X, Y, and Z directions generated by ADAMS.

**Figure 9:**
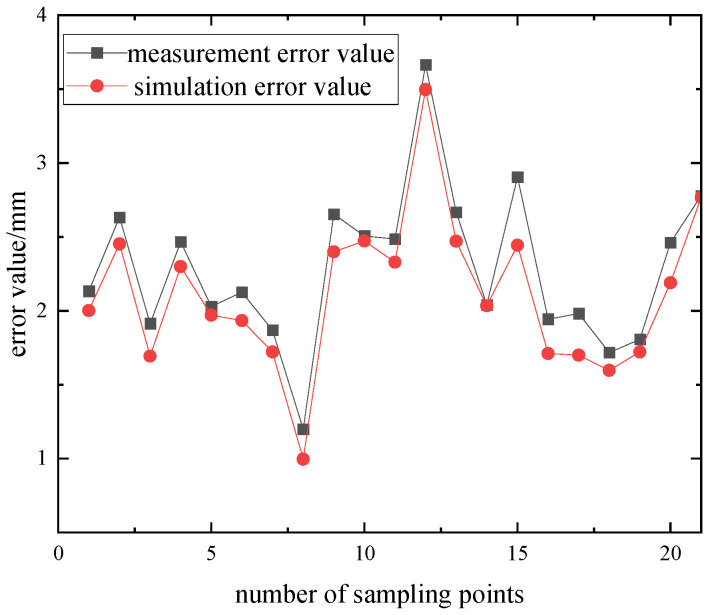
Error value comparison.

As shown in Figure 8, the displacement curves of the robotic arm’s end-effector in the X, Y, and Z directions are plotted from the initial position [0, 0, 0, 0, 0, 0] to the target position [90, 45, 30, 10, 30, 90]. The blue solid line represents the theoretical displacement curve, while the red dashed line represents the displacement curve under the combined effect of the robotic arm’s self-weight and external load. From Figure 9, it can be observed that there is an error between the theoretical and actual displacement curves in the X, Y, and Z directions of the robotic arm’s end-effector.

### 3.3. Error Validation

To validate the effectiveness of the simulation error, the actual position error of the robotic arm’s end-effector was measured, and the error curves for both are shown in Figure 9. From the figure, it can be observed that the actual end-effector error is greater than the ADAMS simulation error. This is because the sources of the robotic arm’s overall motion error are more complex. In addition to kinematic errors caused by factors such as flexible joints, flexible links, and measurement system errors, the errors are also significantly influenced by non-kinematic factors, including assembly errors, joint motor characteristics, and vibrations at the end-effector during startup and operation. The combined effect of these factors results in a larger overall motion error for the actual robotic arm compared to the kinematic simulation error based on ADAMS. However, a comparative analysis of the actual measurement data and simulation data reveals a high degree of consistency in the overall error trends. Therefore, the rigid–flexible coupled robotic arm model is capable of accurately reflecting the error variation patterns of the actual robotic arm, and it can serve as an effective approximation tool for studying the kinematic errors of real robotic arms and their compensation methods.

### 3.4. Collection of Spatial Point Position Errors

**Figure 10:**
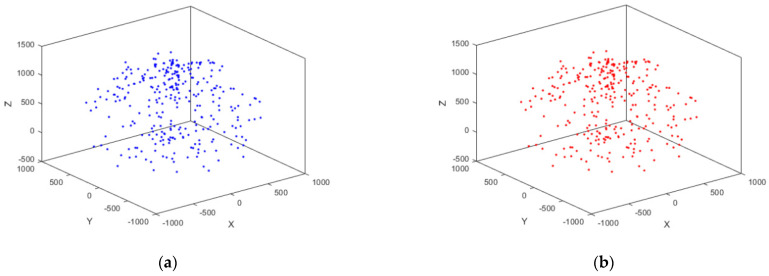
Robotic arm spatial sampling points: (**a**) distribution of theoretical sampling points; (**b**) distribution of simulation sampling points.

Within the robotic arm’s workspace, 300 target points were generated using random sampling, the inverse kinematics method was then used to calculate the joint angles corresponding to each target point. These 300 sets of joint angles were sequentially imported into the ADAMS rigid–flexible coupled robotic arm model for simulation. As a result, 300 sets of end-effector position data, including deviations, were obtained. A portion of this data is presented in Table 2, and the distribution of the spatial sampling points is shown in Figure 10.

**Figure 11:**
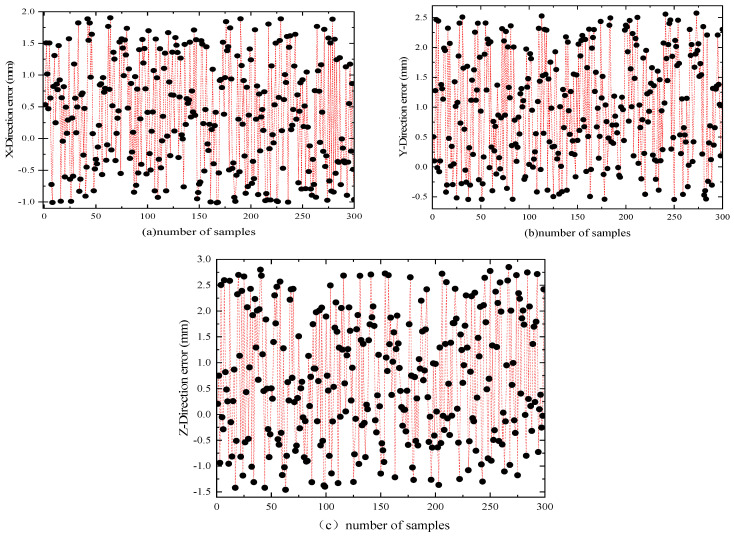
Error values in the X, Y, and Z directions.

By performing interpolation between the target theoretical positions and the simulated positions in the X, Y, and Z directions, the error values in each direction can be calculated. The position errors in these three directions are shown in Figure 11.

## 4. ECOA-BP Error Prediction Model

### 4.1. BP Neural Network

**Figure 12:**
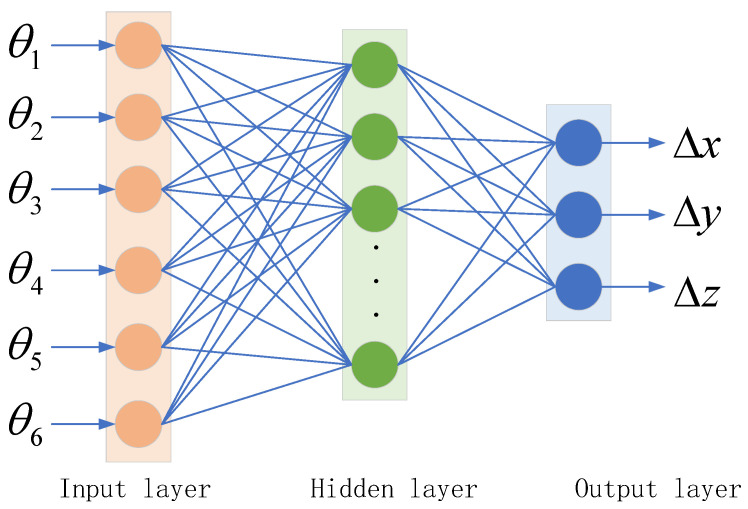
BP neural network structure.

Based on the significant nonlinear characteristics of the error variation curve, neural networks, especially the Backpropagation (BP) algorithm, can effectively fit irregular and highly nonlinear data. Unlike traditional methods, neural networks do not require an explicit mathematical model or equation to derive the error; instead, they automatically learn and construct the error model through training on a large number of error sample data. Therefore, when training and predicting errors using the BP algorithm, the six joint angles of the manipulator [$\theta_{1}$, $\theta_{2}$, $\theta_{3}$, $\theta_{4}$, $\theta_{5}$, $\theta_{6}$] are used as input features, and the errors between the theoretical and actual positions [Δx, Δy, Δz] are used as outputs. The model is trained through the neural network to predict the position errors of the end-effector in the X, Y, and Z directions [24]. The structure of the BP neural network is shown in Figure 12.

As shown in Equation (5), the calculation principle for the neuron input net is as follows:

(5) $$net_{j}=\sum_{i=1}^{n}w_{ij}x_{i},\;j=1,2,\ldots q$$

where $w_{ij}$ represents the weight between the i-th neuron and the j-th hidden-layer neuron; $x_{i}$ is the input value of the neuron; and q is the number of neurons in the hidden layer.

As shown in Equations (6) and (7), the weights and biases are updated in reverse according to the optimization algorithm, iterating continuously until the loss function converges or a certain number of iterations is reached.

(6) $$\Delta w_{ij}=\eta (d_{j}-y_{j})f^{\prime }(net_{j})x_{i}$$

(7) $$w_{ij}(k+1)=w_{ij}(k)+\Delta w_{ij}(k)$$

where η is the learning rate, and k is the number of iterations. $w_{ij}^{l}$ is the connection weight between the j-th neuron of the l−1-th layer and the i-th neuron of the l-th layer, and $b_{j}^{l}$ is the bias of the j-th neuron in the l-th layer.

Finally, the output of the hidden layer neuron is given by Equation (8).

(8) $$net_{j}^{l}=f(\sum_{i=1}^{n}w_{ij}^{l}\cdot net_{j}^{l-1}+b_{j}^{l})$$

where $net_{j}^{l}$ is the output of the j-th neuron in the l−1-th layer, and f(⋅) is the activation function.

### 4.2. Crayfish Optimization Algorithm

In the training process of neural network models, parameter optimization is a key factor that determines the accuracy of the model. However, traditional gradient descent methods are prone to becoming stuck in local minima during the optimization process, which prevents the model parameters from converging to the global optimum and thus limits the improvement of model performance. To overcome this limitation, the Crayfish Optimization Algorithm (COA) is introduced as the core optimization method [25]. The Crayfish Optimization Algorithm, by simulating the foraging behavior and adaptive selection mechanism of crayfish populations, effectively avoids the problem of local minima in complex, high-dimensional optimization spaces, allowing for a more precise search for the global optimum.

The Crayfish Optimization Algorithm was designed based on the ecological behavior of crayfish, with its core mechanism inspired by the regulation of environmental temperature. In the algorithm, the environmental temperature (temp) is set as a random variable to simulate the uncertainty of the environment. The specific formula is as follows:

(9) temp=rand×15+20

(10) $$p=C_{1}\times \left(\frac{1}{\sqrt{2}\times \pi \times \sigma }\times \exp\left(-\frac{{(\mathrm{temp}-\mu )}^{2}}{2\sigma^{2}}\right)\right)$$

where rand is a random number between 0 and 1, μ represents the most suitable environmental temperature for the crayfish, $C_{1}$ is a constant (0.2), and σ is another constant (3). The behavior of the crayfish is divided into three stages based on temperature: the sheltering phase, the competition phase, and the foraging phase. The dynamic switching of behaviors reflects the natural adaptation of crayfish to their environment, providing a balance between global search and local exploitation in the algorithm.

When the temperature is too high, i.e., temp>30 ℃, crayfish tend to seek shelter in caves to avoid the heat. The definition of the shaded cave $X_{\mathrm{shade}}$ is as follows:

(11) $$X_{\mathrm{shade}}=\left(X_{G}+X_{L}\right)/2$$

where $X_{G}$ represents the optimal position obtained through the number of iterations, and $X_{L}$ represents the optimal position obtained after updating the previous generation of the population. In the sheltering phase, the competition for caves is a random event.

When rand<0.5, it indicates that the cave resource has not been occupied by other crayfish, and the crayfish will enter the cave to shelter from the heat. The process of entering the cave for shelter is shown in the following formula:

(12) $$X_{i,j}^{t+1}=X_{i,j}^{t}+C_{2}\times rand\times (X_{shade}-X_{i,j}^{t})$$

where t represents the current iteration, t+1 represents the next generation iteration, $X_{i,j}^{t}$ is the position of the i-th crayfish in the t-th iteration, and $C_{2}$ is a decreasing curve, calculated as follows:

(13) $$C_{2}=2-(t/T)$$

where T represents the maximum number of iterations.

When cave resources are limited, multiple crayfish compete for the opportunity to enter the cave. This behavior corresponds to the competition phase of the algorithm, where the interaction between crayfish individuals is simulated to achieve optimal resource allocation. When the environmental temperature temp>30 ℃, and rand≥0.5, it indicates that other crayfish have also chosen the same cave. In this case, they will compete for the cave, and each crayfish will adjust its position based on the position of another crayfish, as shown in the following formula:

(14) $$X_{i,j}^{t+1}=X_{i,j}^{t}-X_{z,j}^{t}+X_{shade}$$

(15) z=round(rand×(N−1))+1

where N represents the crayfish population size, and z represents a random individual within the crayfish population.

When temp≤30 ℃, the crayfish enter the foraging phase. The food position and food size are defined as follows:

(16) $$X_{food}=X_{G}$$

(17) $$Q=C_{3}\times \mathrm{rand}\times (fitness_{i}/\;fitness_{food})$$

where $C_{3}$ is the food factor, representing the maximum food, with a constant value of 3. $fitness_{i}$ represents the fitness value of the i-th crayfish, and $fitness_{food}$ represents the fitness value at the food location.

When $Q>(C_{3}+1)/2$, it indicates that the food is too large, and the crayfish will tear the food apart using the following formula:

(18) $$X_{food}=\exp(-\frac{1}{Q})\times X_{food}$$

After the food becomes smaller, the crayfish will use its pincers to tear the food apart and then alternate between the second and third claws to grab and eat the food. In the algorithm, this alternating process is simulated using a combination of sine and cosine functions, as shown in the following formula:

(19) $$X_{i,j}^{t+1}=X_{i,j}^{t}+X_{food}\times p\times (\cos(2\times \pi \times rand)-\sin(2\times \pi \times rand))$$

When $Q\leq (C_{3}+1)/2$, it indicates that the food is not too large, and the crayfish will move directly toward the food and consume it. The equation is as follows:

(20) $$X_{i,j}^{t+1}=(X_{i,j}^{t}-X_{food})\times p+p\times rand\times X_{i,j}^{t}$$

### 4.3. Improved Crayfish Optimization Algorithm (ECOA)

Although the Crayfish Optimization Algorithm (COA) performs well in various optimization problems, it still has limitations when dealing with complex, high-dimensional, or multi-modal problems. During the search process, the population may converge prematurely to local optima due to a lack of diversity. Additionally, the update rules in the sheltering and foraging phases are relatively simple and may not fully explore the search space. In high-dimensional problems, the algorithm’s convergence speed may also be suboptimal. To address these issues, this paper proposed improvements to the COA, aiming to enhance its global search ability, accelerate convergence, and avoid becoming trapped in local optima.

(1) Mirror Reflection Learning

Mirror Reflection Learning enhances the diversity of the population by generating the mirror positions of individuals in the current population, helping to avoid the algorithm from becoming trapped in local optima. This mechanism simulates the reflection behavior of crayfish in nature when they encounter obstacles, providing additional search directions and expanding the search space. For each individual $X_{i}$ in the population, its mirror reflection position $X_{MRL}$ is calculated as follows:

(21) $$X_{MRL}=(0.5+0.5\lambda )\;(X_{\min}+X_{\max})+\lambda X_{i}$$

where $X_{\min}$ and $X_{\max}$ represent the minimum and maximum values in each dimension of the current population, respectively, and λ is a random number in the range [0, 2], which is generated as follows:

(22) $$\lambda =\left\{\begin{array}{c} 1+rand^{2}\begin{array}{c} \;\;\;\;\;if\;rand<0.5 \end{array}\\ 1-rand^{2}\begin{array}{c} \;\;\;\;\;if\;rand\geq 0.5 \end{array} \end{array}\right.$$

(2) Integration of the Aquila Optimizer (AO) in the Sheltering Phase

In the original COA, the update rules during the sheltering phase are relatively simple, which may not be sufficient to effectively guide the population toward the global optimum. By integrating the strategy of the Aquila Optimizer (AO), a more guided update mechanism was introduced to improve the algorithm’s convergence speed and search accuracy. This integration provides a more efficient and focused search process, helping the algorithm converge more quickly while enhancing the precision of the solution.

(23) $$X_{i,j}^{t+1}=X_{food}(1-\frac{t}{T})+(\frac{\sum_{i=1}^{N}X_{i}}{N}-X_{food})\cdot rand$$

where t is the current iteration number, and T is the maximum number of iterations

(3) Arithmetic Crossover

Arithmetic crossover increases the genetic diversity of the population by exchanging information between different dimensions, thereby expanding the search space. This enhances the global search capability and helps prevent the algorithm from becoming trapped in local optima.

For each individual $X_{i}$ in the population, two dimensions $index_{1}$ and $index_{2}$ are randomly selected, and a linear combination is performed to generate a new individual position $X_{MSvi}$. The formula for generating $X_{MSvi}$ is as follows:

(24) $$X_{MSvi,j}=rand\cdot X_{i,index_{1}}+(1-rand)\cdot X_{i,index_{2}}$$

(4) Introduction of Levy Flight

Levy flight is a random walk strategy characterized by long-distance jumps, which enables effective exploration of the search space and helps the algorithm escape from local optima. By introducing Levy flight, the global search ability of the algorithm is significantly enhanced.

(25) $$X_{i,j}^{t+1}=(X_{i,j}^{t}-X_{food})\times p+p⊗L\mathrm{evy}(\lambda )\times X_{i,j}^{t}$$

**Figure 13:**
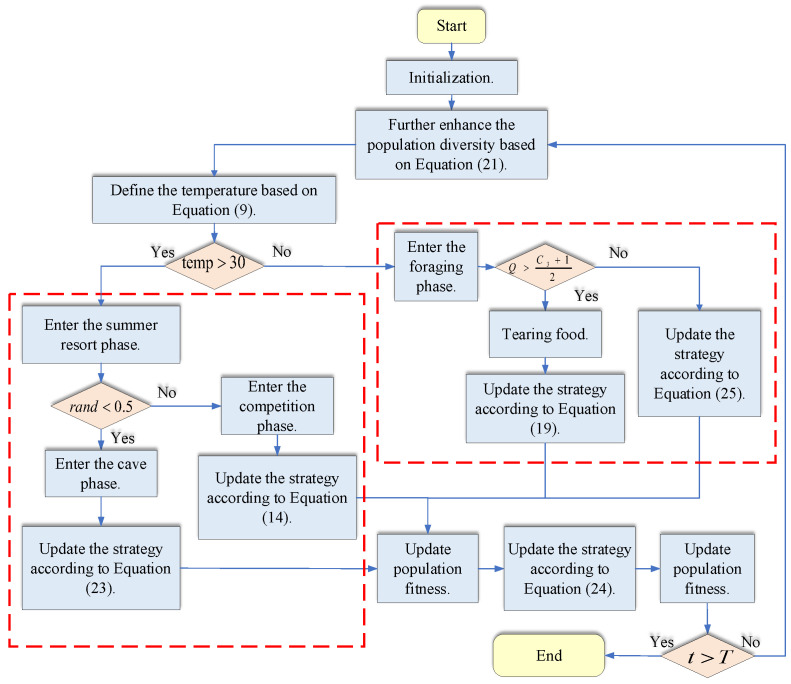
Flowchart of the ECOA optimization algorithm.

The operational flow of the above ECOA is shown in Figure 13.

### 4.4. Performance Testing of the Improved Algorithm

This study compared the performance of several efficient optimization algorithms, including the Whale Optimization Algorithm (WOA), Marine Predators Algorithm (MPA), Harris Hawks Optimization (HHO), Crayfish Optimization Algorithm (COA), Dung Beetle Optimization (DBO), Grey Wolf Optimization Algorithm (GWO), and Enhanced Crayfish Optimization Algorithm (ECOA). To ensure fairness and accuracy in the comparison, all algorithms were evaluated using the same test functions. During the experiment, the test functions listed in Table 3 were run independently 30 times to ensure that the results were statistically representative. Additionally, the parameters for each run were kept consistent. The experiment was set with T=500 iterations and a population size of N=30, providing a direct comparison of the optimization performance of the different algorithms.

**Figure 14:**
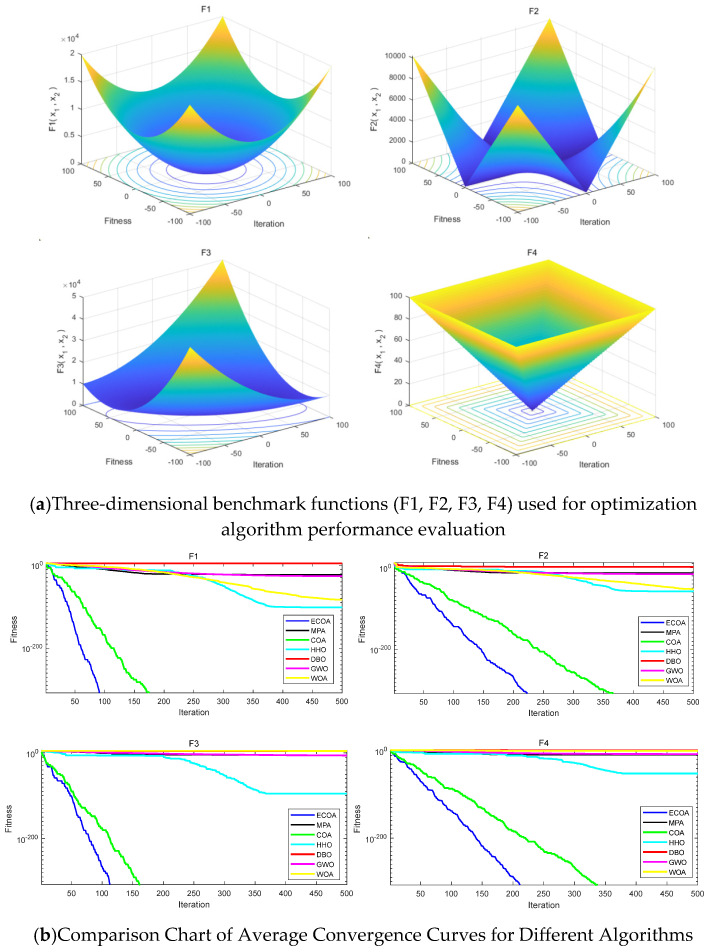
Algorithm performance comparison.

Figure 14 shows the comparison of average fitness curves obtained from running four test functions using the Enhanced Crayfish Optimization Algorithm (ECOA). From the figure, it can be observed that the ECOA is able to find the global optimum more quickly and accurately compared to other algorithms. The comparison results demonstrate that the improved algorithm performs excellently in both global search and local search capabilities.

### 4.5. Simulation Experiment on End-Effector Positioning Error Compensation of Robotic Arm

**Figure 15:**
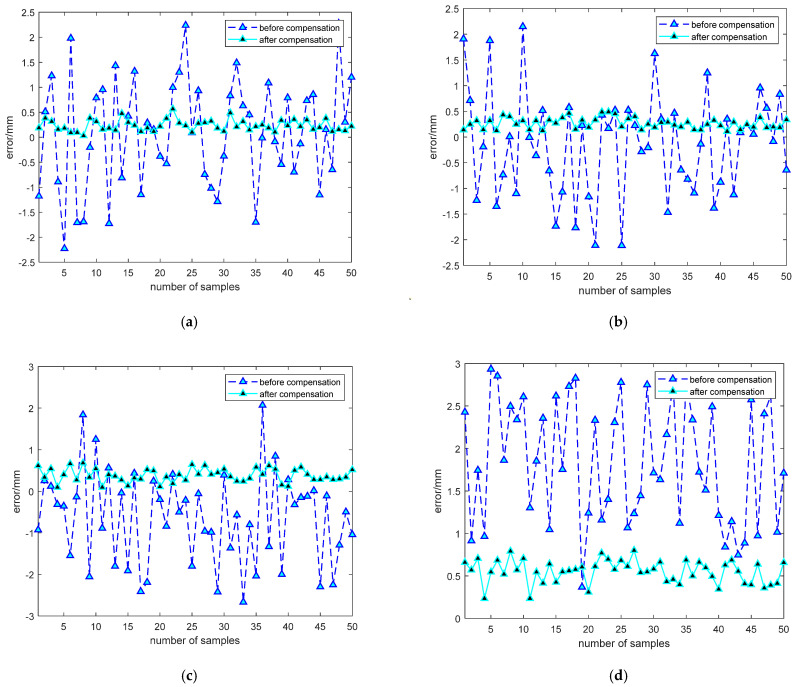
Error compensation simulation results: (**a**) comparison of X-direction errors before and after compensation; (**b**) comparison of Y-direction errors before and after compensation; (**c**) comparison of Z-direction errors before and after compensation; (**d**) comparison of absolute positioning error before and after compensation.

The MATLAB-based simulation experiment for compensating the end-effector positioning error of an industrial robot used 300 error samples, which were divided into a training set and a test set at a 5:1 ratio. The test set data were used to validate the neural network, and the effectiveness of the compensation method was assessed by comparing the average positioning errors in the X, Y, and Z directions and the absolute positioning error before and after compensation. The experimental results show that the end-effector positioning error is significantly reduced after compensation, validating the effectiveness of the ECOA-BP neural network compensation model. As shown in Figure 15, before compensation, the average positioning errors in the X, Y, and Z directions and the absolute positioning average error were 0.75 mm, 0.94 mm, 1.26 mm, and 1.71 mm, respectively, in the test set data. After compensation, the average positioning errors in the X, Y, and Z directions and the absolute positioning average error were reduced to 0.21 mm, 0.24 mm, 0.39 mm, and 0.52 mm, respectively. These results demonstrate that the ECOA-BP neural network compensation method can effectively reduce the end-effector positioning error and significantly improve the robot’s positioning accuracy in complex environments.

### 4.6. Model Comparison and Validation Experiment

**Figure 16:**
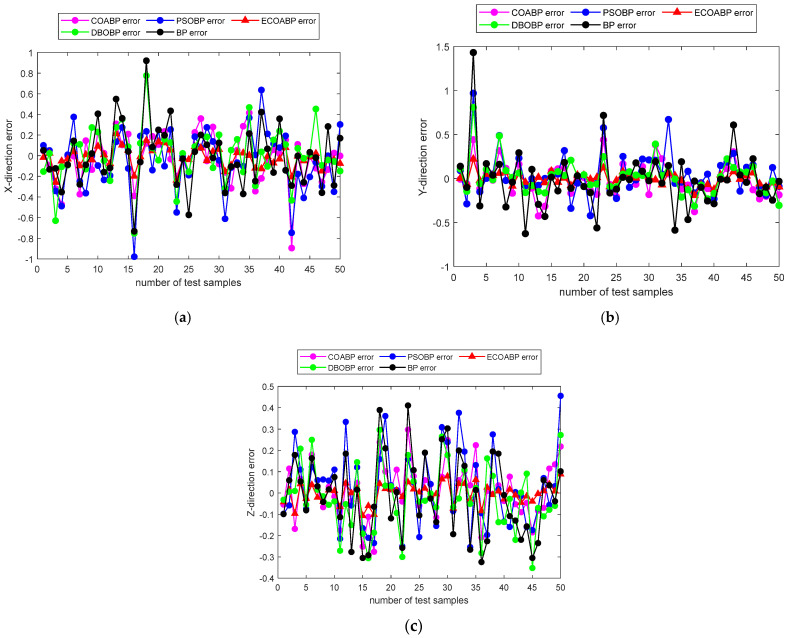
Comparison of predicted errors in the X, Y, and Z directions: (**a**) prediction errors in the X direction for various algorithms; (**b**) prediction errors in the Y direction for various algorithms; (**c**) prediction errors in the Z Direction for various algorithms.

To validate the superiority of the ECOA-BP model proposed in this paper for end-effector positioning error compensation in industrial robots, a comparative experiment was conducted with four other models: DBO-BP [26], PSO-BP [27], BP, and COA-BP. As shown in Figure 16a–c, the errors in the X, Y, and Z directions for each model are presented, with the red triangles representing the errors of ECOA-BP. The results show that ECOA-BP exhibits the smallest errors among all models, demonstrating its significant advantage in error compensation.

**Figure 17:**
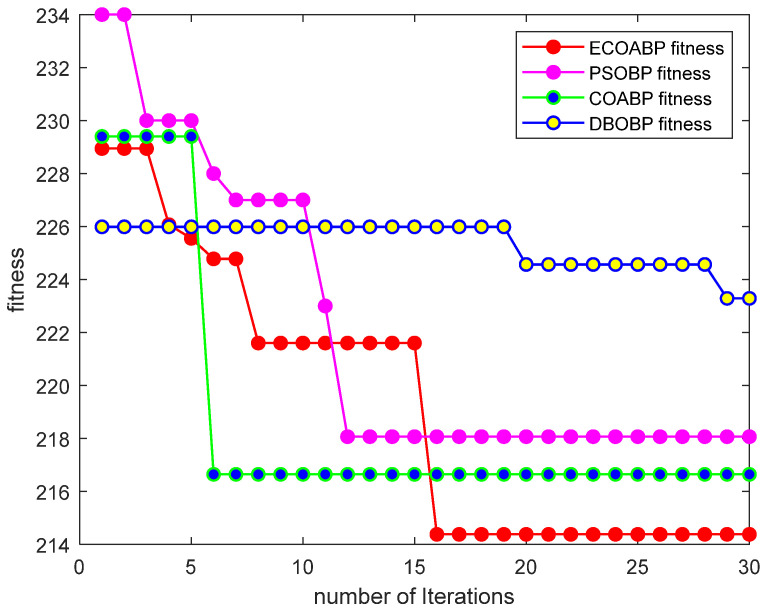
Iteration plot of four algorithms optimizing BP neural network.

As shown in Figure 17, the fitness curve of the ECOA-BP model demonstrates significant convergence during the iterations, reaching a lower fitness value in fewer iterations. This indicates that the ECOA-BP model can quickly converge to the optimal solution, exhibiting higher accuracy and stability.

## 5. Experimental Verification

### 5.1. Experimental Platform Setup

**Figure 18:**
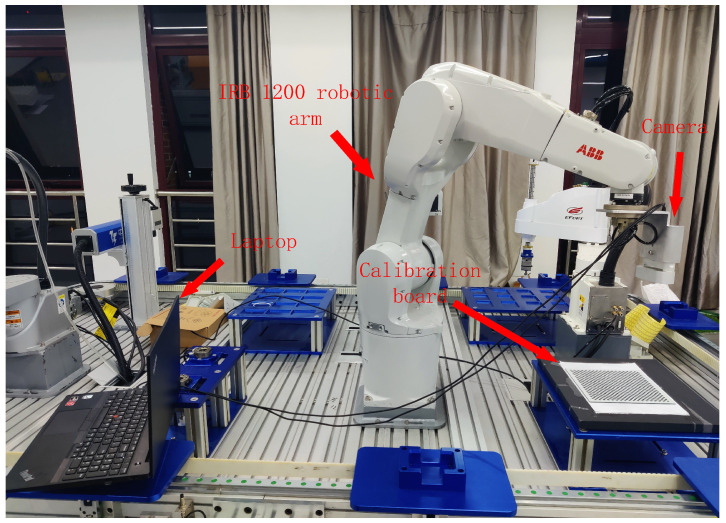
Experimental platform.

To validate the positioning error compensation method proposed in this paper, an experimental platform was established, as shown in Figure 18. The platform consists primarily of an IRB1200-5/0.9 industrial robot, a visual camera, a black and white checkerboard calibration plate, and a laptop. The IRB1200 robot serves as the core component of the experiment, with a working range of 901 mm, repeatability of 0.025 mm, and an effective payload capacity of 5 kg. The visual camera used is an Azure Kinect camera, which features an RGB camera and a 1-megapixel time-of-flight (ToF) depth sensor. The camera’s resolution is 3840 × 2160, with a working range from 0.5 to 3.86 m, which is utilized for measuring the robot’s pose. The black and white checkerboard calibration plate has a size of 9 × 12, with each square measuring 20 mm. The manufacturing tolerance of the calibration plate is ±0.01 mm, and the accuracy of the corner point location is controlled within ±0.005 mm. By using this calibration plate, chessboard corner points can be accurately extracted for both camera calibration and hand-eye calibration between the robot and the camera.

In the experiment, the camera was fixed at the end-effector of the robotic arm, while the calibration plate was placed at a fixed location. As the robotic arm moved to the target position under the control of the teach pendant, the visual camera captured images of the calibration plate to obtain the current position information of the robot. During the experiment, the ambient temperature was maintained between 18 °C and 26 °C, and electromagnetic interference was minimized to avoid any impact on the experimental results. Through precise calibration and multiple measurements, the repeatability and reliability of the experiment were ensured.

### 5.2. IRB1200 Robot Arm End-Effector Positioning Error Compensation Experiment

Using the experimental platform described above, error compensation experiments were conducted for 50 target points. First, the actual positions corresponding to each target point and the theoretical positions were collected and calculated. The improved ECOA-BP algorithm was used to predict the position errors of the end-effector in the X, Y, and Z directions. The pseudo-target point method [28,29] was then used to calculate the optimized joint angles, and the corresponding joint angle adjustments were determined. These computed joint angle adjustments were input into the robot’s controller, and the actual position of the robot’s end-effector under the suspended load condition was measured. Finally, the position errors in the X, Y, and Z directions and the absolute positioning error were calculated.

**Figure 19:**
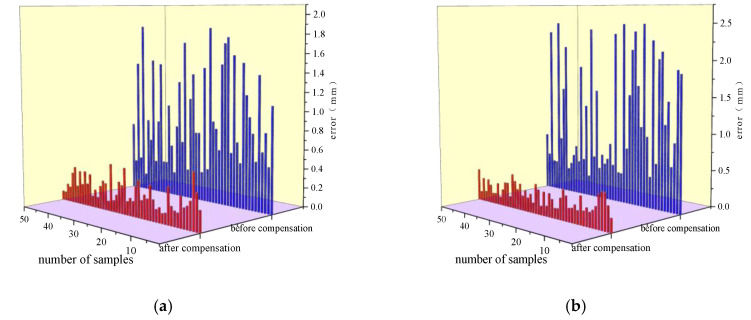

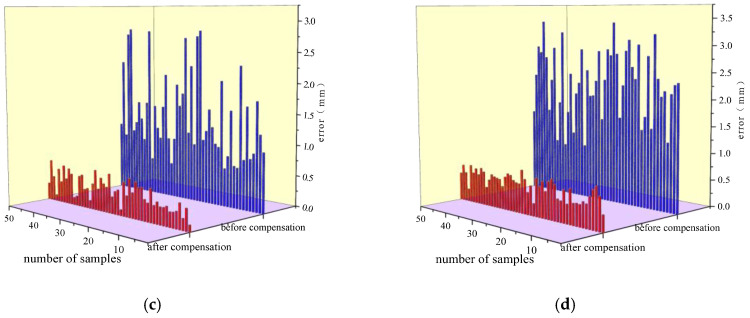
Comparison of errors before and after compensation: (**a**) comparison of X-direction errors before and after compensation; (**b**) comparison of Y-direction errors before and after compensation; (**c**) comparison of Z-direction errors before and after compensation; (**d**) comparison of absolute positioning error before and after compensation.

The data before and after compensation are shown in Table 4. The experimental results, based on 50 sets of data, are presented in Figure 19, where (a), (b), (c), and (d) show detailed comparisons of the robot’s position errors in the X, Y, Z directions and the absolute positioning error before and after compensation. There is a significant difference in the errors before and after compensation. Before compensation, the average errors in the X, Y, and Z directions were 0.9 mm, 1.19 mm, and 1.45 mm, respectively. After compensation, the average errors in the three directions were reduced to 0.24 mm, 0.26 mm, and 0.38 mm. The absolute positioning error decreased from 2.27 mm to 0.55 mm, resulting in a 75.77% improvement in absolute positioning accuracy. The experimental results demonstrate that the method proposed in this paper can effectively compensate for the positioning errors of industrial robots, significantly improving their positioning accuracy.

### 5.3. Performance of the Compensation Method Under Different Load Conditions

In practical applications, industrial robots are often required to operate under varying load conditions, and changes in load can impact the robot’s motion precision and positioning accuracy. To evaluate the performance of the proposed end-effector positioning error compensation method based on the ECOA-BP neural network under different load conditions, this section presents compensation experiments on the positioning errors of 50 target points, tested under 1 kg and 5 kg load conditions. For each load condition, 50 target points were tested, and the absolute positioning errors of the robot’s end-effector were calculated before and after compensation. By adjusting the load and repeating the same experimental steps, the adaptability and robustness of the compensation method under different load conditions were ensured.

**Figure 20:**
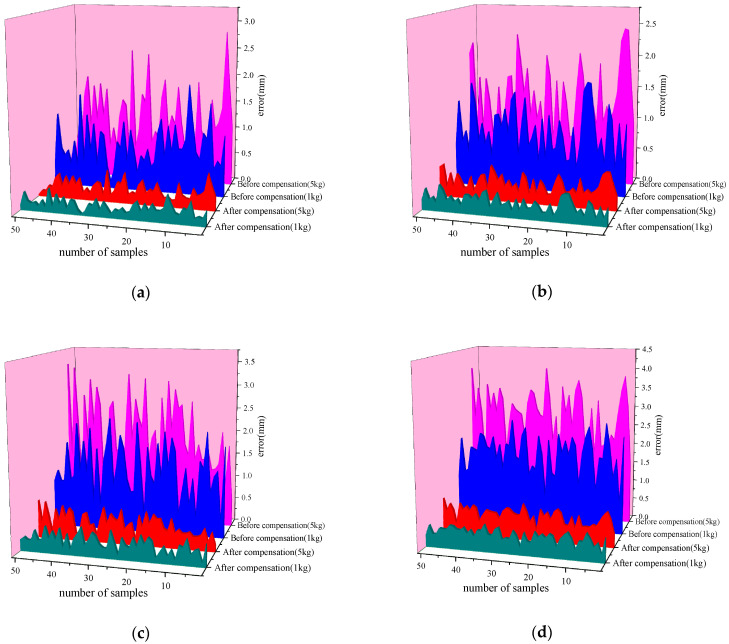
Compensation comparison under different load conditions: (**a**) comparison of the X-direction error before and after compensation under different load conditions; (**b**) comparison of the Y-direction error before and after compensation under different load conditions; (**c**) comparison of the Z-direction error before and after compensation under different load conditions; (**d**) comparison of the absolute error before and after compensation under different load conditions.

As shown in Figure 20, before compensation, the average absolute positioning error for the 1 kg load was 1.95 mm, while for the 5 kg load, it was 2.78 mm. After compensation, the average absolute positioning error was reduced to 0.47 mm for the 1 kg load and 0.64 mm for the 5 kg load. The absolute positioning accuracy improved by 75.89% for the 1 kg load and 76.97% for the 5 kg load. These results demonstrate that although the positioning errors increased with the load, the ECOA-BP neural network-based compensation method significantly improved positioning accuracy under both 1 kg and 5 kg load conditions. This indicates that the proposed method is not only effective for compensating end-effector positioning errors under varying load conditions but also exhibits strong adaptability and robustness.

## 6. Discussion

This study proposed an industrial robot end-effector position error compensation method based on the Enhanced Crayfish Optimization Algorithm (ECOA) combined with BP neural networks (ECOA-BP), and its effectiveness and reliability was verified through experiments. The experimental results show that the proposed ECOA-BP model significantly improves the positioning accuracy of industrial robots, particularly in compensating for flexible errors, achieving better performance than traditional methods.

First, addressing the errors caused by factors such as joint flexibility, link deformation, and end-effector load in industrial robots during real-world applications, this study established a kinematic model of the robot based on the DH parameter method. The model was combined with ANSYS and ADAMS simulation platforms to successfully build a rigid–flexible coupled virtual prototype model. Through simulation analysis of the virtual prototype, the variation in the robot’s end-effector position error was obtained. Based on this, experimental data were used for error compensation research, and a compensation method combining the Enhanced Crayfish Optimization Algorithm (ECOA) and BP neural networks was proposed.

Compared to traditional gradient descent methods, the ECOA can effectively avoid local optima and, by introducing techniques such as Mirror Reflection Learning, Aquila Optimizer, and Levy flight, enhances global search ability and convergence speed. This significantly improves the accuracy of the error compensation model. By comparing the compensation effects of different optimization algorithms (such as COA, PSO, and DBO) combined with BP neural networks, the ECOA-BP model shows a significant advantage in compensating for end-effector position errors, especially in addressing complex, high-dimensional error data.

The experimental verification results indicated that the ECOA-BP-based compensation method successfully reduced the end-effector positioning errors. Before compensation, the average errors in the X, Y, and Z directions were 0.9 mm, 1.19 mm, and 1.45 mm, respectively. After compensation, the average errors in the three directions were reduced to 0.24 mm, 0.26 mm, and 0.38 mm, respectively. The absolute positioning error decreased from 2.27 mm to 0.55 mm, improving positioning accuracy by 75.77%. These results show that the proposed method can effectively improve the robot’s positioning accuracy and has high practical application value.

## 7. Conclusions

This paper proposed an industrial robot end-effector position error compensation method based on the Enhanced Crayfish Optimization Algorithm (ECOA) combined with BP neural networks (ECOA-BP), and its effectiveness was verified through experiments. This method combined virtual prototype modeling and optimization algorithms, significantly improving the robot’s positioning accuracy, especially in addressing flexible error compensation issues. The experimental results demonstrated that the ECOA-BP error compensation model shows a significant advantage in reducing the end-effector positioning errors of the robot. The average errors in the X, Y, and Z directions were reduced to 0.24 mm, 0.26 mm, and 0.38 mm, respectively, and the absolute positioning error decreased by 75.77%, significantly improving the robot’s positioning precision. This study not only provided an effective compensation solution for improving the accuracy of industrial robots but also offered a theoretical foundation and practical guidance for future research on similar error compensation problems.

## Figures and Tables

**Table 1:** DH parameters of the IRB1200 robotic arm.

Connecting Rod	$\theta_{i}$ (°)	$d_{i}$ (mm)	$a_{i}$ (mm)	αi (°)
1	$\theta_{1}$	399.1	0	−90
2	$\theta_{2}$	0	448	0
3	$\theta_{3}$	0	42	−90
4	$\theta_{4}$	451	0	90
5	$\theta_{5}$	0	0	−90
6	$\theta_{6}$	82	0	0

**Table 2:** Position data collection.

Theoretical Position			Error Position		
X	Y	Z	Xe	Ye	Ze
49.42691845	−954.7765678	451.2838061	48.1858784	−952.7678152	450.3083355
−14.71450535	−3.145353164	572.3243259	−14.17841629	−2.394533308	572.5949185
−28.50114285	138.9999386	99.30782722	−27.20689011	137.7030787	99.43791213
47.99561916	−20.3883509	220.4096536	47.05575017	−20.59107919	220.0787374
786.5074399	−203.1581306	561.2340634	784.1631597	−201.1819812	560.8600316
−19.6647607	726.3016702	809.3148585	−17.58017033	724.880572	807.6861587
195.2609027	357.2285938	565.1706256	193.4641369	356.4558447	565.0305278
127.7424791	248.9245901	−337.8192471	125.9627131	248.9303179	−335.8854424
−100.1742408	62.99946724	1264.307026	−100.3888537	61.84046532	1262.143111
−102.2394238	−894.6917867	123.0291653	−101.4104325	−892.4307883	124.3466362
−44.29791296	356.7232431	1039.530697	−43.29593407	356.7161797	1038.596011
49.24616674	276.0077411	269.0172772	47.42788198	275.6254632	269.6122395
−111.8532794	−166.9370591	1340.115526	−110.3469053	−166.3933479	1338.22094
−136.1207034	211.4090006	265.5810421	−136.9758344	210.7181641	265.5399275

**Table 3:** Test functions.

Test Function	Dimension	Boundary Conditions
$F_{1}(x)=\sum_{i=1}^{n}x_{i}^{2}$	30	[−100, 100]
$F_{2}(x)=\sum_{i=1}^{n}\left.\left|x_{i}\right.\right|+\prod_{i=1}^{n}\left.\left|x_{i}\right.\right|$	30	[−10, 10]
$F_{3}(x)=\sum_{i=1}^{n}(\sum_{j-1}^{i}x_{j}{)}^{2}$	30	[−100, 100]
$F_{4}(x)=\max_{i}\left\{\left|\left.x_{i}\right|\right.,1\leq x_{i}\leq n\right\}$	30	[−100, 100]

**Table 4:** Comparison of measured values before and after compensation.

	Theoretical Position (mm)			Measured Position (mm)			Compensated Position (mm)		
	X0	Y0	Z0	X1	Y1	Z1	X2	Y2	Z2
1	35.14	302.4	762.3	36.06	304.06	764.03	35.36	302.59	762.41
2	35.17	376.58	665.47	35.86	378.73	666.77	35.55	376.91	665.82
3	101.8	419.68	661.6	101.95	419.96	661.15	102.37	420.17	661.79
4	−2.45	456.56	635.69	−0.62	457.09	635.65	−2.16	457.05	636.10
5	138.74	312.72	693.86	138.45	314. 31	693.76	138.97	313.17	694.13
6	114.18	387.03	620.28	114.53	387.46	620.68	114.39	387.32	620.53
7	96.62	589.96	636.1	96.97	592.53	637.34	96.935	590.19	636.34
8	43.27	622.35	572.86	43.39	623.40	574.69	43.42	622.55	573.17
9	43.28	561.84	626.67	42.42	562.87	627.87	43.44	562.09	626.96
10	101.71	605.01	639.86	103.00	604.75	640.42	101.91	605.21	640.14

## Data Availability

The data used to support the findings of this study are available from the corresponding author upon request.
